# Causes and Consequences of Not Having a Personal Healthcare Provider Among American Indian Elders: A Mixed-Method Study

**DOI:** 10.3389/fpubh.2022.832626

**Published:** 2022-03-02

**Authors:** Elise Trott Jaramillo, David H. Sommerfeld, Emily A. Haozous, Amy Brunner, Cathleen E. Willging

**Affiliations:** ^1^Southwest Center, Pacific Institute for Research and Evaluation, Albuquerque, NM, United States; ^2^Department of Psychiatry, University of California, San Diego, San Diego, CA, United States

**Keywords:** American Indians, health equity, healthcare utilization, continuity of care, health policy

## Abstract

Having a regular relationship with a healthcare provider contributes to better health outcomes and greater satisfaction with care for older adults. Although members of federally recognized American Indian tribes have a legal right to healthcare, American Indian Elders experience inequities in healthcare access that may compromise their ability to establish a relationship with a healthcare provider. This multi-year, community-driven, mixed-method study examines the potential causes and consequences of not having a personal healthcare provider among American Indian Elders. Quantitative surveys and qualitative interviews were conducted with 96 American Indian Elders (age 55 and over) in two states in the Southwestern United States. Quantitative and qualitative data were analyzed separately and then triangulated to identify convergences and divergences in data. Findings confirmed that having a consistent healthcare provider correlated significantly with self-rated measures of health, confidence in getting needed care, access to overall healthcare, and satisfaction with care. Lack of a regular healthcare provider was related to interconnected experiences of self-reliance, bureaucratic and contextual barriers to care, and sentiments of fear and mistrust based in previous interactions with medical care. Increasing health equity for American Indian Elders will thus require tailored outreach and system change efforts to increase continuity of care and provider longevity within health systems and build Elders' trust and confidence in healthcare providers.

## Introduction

Having a regular and reliable relationship with a healthcare provider is strongly associated with more use of preventive care, greater satisfaction with care, lower healthcare costs, and better health outcomes (1–5). This is particularly true for older adults, among whom an ongoing relationship with a provider is associated with lower mortality and reduced risk of preventable hospitalization (6–8). In contrast, the consequences of not having a healthcare provider include more hospitalizations and unmet needs, longer hospital stays, problems with medications, and poorer health outcomes (4, 5). Many of the ~25% of all adults in the United States (U.S.) who do not have a personal healthcare provider report that this is because they are seldom sick and do not have many healthcare-related needs (4, 9). However, racial/ethnic minority status, a lack of health insurance, and the cost of medical care (with or without insurance) are common risks that correlate with the lack of a personal healthcare provider (9). There is little research examining this lack among American Indians. To our knowledge, no studies have assessed the causes and consequences of not having a personal healthcare provider for American Indian Elders, a rapidly growing population that has a greater likelihood of poor health and barriers to care than any other aging group in the U.S. (10–12). This research addresses that gap.

Members of federally recognized American Indian tribes have a legal right to healthcare that has been guaranteed by treaties with the U.S. government, the U.S. Constitution, and the Supreme Court (13). Tribal members can receive available healthcare services at an Indian Health Service (IHS) or tribally run facility in their tribe's service area at no cost and without health insurance. The Purchased/Referred Care (PRC) program provides referrals for services that are not available at the IHS. However, despite the guarantee of healthcare, research describes enduring inequities in health and access to care for this population (10). Unlike Medicaid and Medicare, funding for the IHS budget is appropriated by Congress every year and it consistently falls far short of needs (13), leading to chronic problems with outdated facilities and high rates of provider turnover and vacancies (14). Moreover, although the majority of American Indians live in urban areas outside of their IHS service area, <1% of the IHS budget goes to urban clinics (15).

Although American Indian Elders are likely to have access to care outside the IHS through Medicaid and/or Medicare coverage, the use of health insurance is made more difficult for them by the predominance of confusing jargon and requirements. Access can also be compromised by geographic distance or limited provider networks (16, 17). These challenges may be compounded by a lack of healthcare and insurance services that are congruent with the knowledge systems and informational needs of this population, which may include low health literacy (i.e., the ability to locate, understand, and use information about health and healthcare) caused by a lack of educational opportunity (18), difficulty using the technology involved in finding health information (19, 20), and problems with information processing caused by impairments in cognition, vision, or hearing (21). Finally, Elders may be deterred from seeking care by experiences of discrimination, poor treatment, and cultural memories of medical atrocities, such as the forced sterilization of American Indian women (22–25). This study examines Elders' challenges in establishing a reliable relationship with a healthcare provider in this context and the potential consequences of lacking one.

## Materials and Methods

This mixed-method analysis examines quantitative survey data and qualitative interview data that were collected as part of a multi-year study exploring experiences with healthcare and health insurance among American Indian Elders (age 55 and older) in two states in the Southwestern U.S. (26). Consistent with community-engaged approaches to conducting research with Indigenous peoples (27, 28), the study was conceptualized and designed by a team of Indigenous and non-Indigenous researchers in consultation with a group of Elders and allies comprising the Seasons of Care Community Action Board (CAB). The CAB members have overseen and participated in each aspect of the research, including reviewing data collection instruments, assisting with recruitment, and strategizing efforts to promote Elder health. Formal approval was obtained from each tribal community where the research was conducted. The study design and written informed consent protocols were reviewed and approved by the Southwest Tribal Institutional Review Board.

### Participants

To recruit Elder participants, we used a purposive sampling strategy designed to represent a range of knowledge, beliefs, and experiences related to healthcare (29). Members of the research team (including the first and fifth authors) conducted in-person outreach during regular visits to American Indian-serving senior centers, health clinics, and meetings of health-focused American Indian groups and organizations. The researchers presented the research study and stayed on-site for several hours to answer questions, screen candidates, and conduct interviews. They also distributed flyers and business cards with a phone number for Elders to call if they wanted to learn more about the study. Eligible participants were age 55 and older and self-identified as American Indian. Although age 65 is more commonly used to define older adults, age 55 more appropriately reflects the cumulative effects of historical trauma on reduced life expectancy among American Indians (30).

A total of 96 Elder participants ranged in age from 55 to 89 years and had an average age of 67 years. A total of 70.8% (n=68) of participants were female. All self-identified as American Indian, and 17.7% (n=17) also reported having Hispanic or Latina/o heritage. Nearly half (47.9%) spoke an Indigenous language as their first language. Twenty-seven percent had a high school diploma/general equivalency diploma or less; 33.3% had some college or vocational training; 22.9% had an associate's or vocational training degree; 9.4% had a bachelor's degree; 5.2% had a master's degree; and 2.1% had a doctoral degree. The Elders came from more than a dozen tribal backgrounds and resided in both reservation and non-reservation settings, including several rural areas and three urban centers.

### Data Collection

Data were collected by experienced researchers with advanced degrees (including the first and fifth authors) paired with Elder consultants from the communities participating in the study. The Elder consultants received training in data collection and human subjects' protections to help researchers conduct interviews and provide cultural and linguistic translations when necessary. Interviews were conducted in English with the Elder consultants available to provide assistance in the Indigenous languages of participants preferring this option. Participants were first asked to complete a quantitative survey gathering information about demographics, health status, health insurance, and healthcare access, utilization, and satisfaction. Researchers then followed a semi-structured interview guide to ask a series of open-ended questions about Elders' perceptions of wellness and experiences with healthcare and health insurance. The guide included questions and probes designed to elicit factors influencing their decisions about help-seeking and care [e.g., who cares for you when you are sick or need help with your health? What is it like to get care at (local facility)?]. Both quantitative and qualitative instruments were developed through iterative review and feedback by members of the CAB regarding the topics to be covered and the wording of questions. This process ensured that the questions were appropriate for Elder participants and that the resulting data were relevant to understanding Elders' experiences. The interviews typically lasted between 60 and 90 min and were conducted in a setting deemed private and convenient by the Elder (e.g., homes, private rooms in senior centers). The qualitative portions of the interviews were digitally recorded and professionally transcribed for analysis.

### Data Analysis

Quantitative survey data were entered into SPSS Version 27. From these data we created the two groups of interest for the present analysis based on participant responses to the following question: “A personal healthcare provider is a provider who knows you well and is familiar with your health history. Do you have someone who you think of as your personal healthcare provider?” Following the formation of the two groups, descriptive and comparative statistics were generated across demographic, health status, healthcare utilization, and health satisfaction domains to identify potential causes and consequences of having or not having a personal healthcare provider.

Qualitative data were analyzed using grounded theory methodology, an iterative approach to analysis that allows findings to emerge from textual data (31). Four team members reviewed the transcripts using Nvivo 12 to develop and agree on a coding scheme that represented the range of issues concerning healthcare for American Indian Elders. Codes were assigned to segments of text ranging from a phrase to several paragraphs based on the coding scheme. Open coding was then used to identify and define new codes that had not previously been considered, followed by focused coding to determine which themes/issues recurred or represented unusual concerns to participants (32). By constantly comparing and contrasting codes with one another, codes with similar content or meaning were grouped into broader themes linked to segments of transcript text (32).

For this analysis, two team members did an additional round of coding on the interviews of Elders who reported in the quantitative survey that they did not have a personal healthcare provider. Additional codes were created based on key sensitizing concepts related to relationships with healthcare providers (e.g., help-seeking, trust, and turnover). These concepts provided “a general sense of reference” and supplied descriptive data based on the words of Elders, enabling us to examine their salience and meaning (29). The researchers created a detailed outline describing and linking codes to each theme/issue and reviewed this work collectively. Discrepancies in coding and analysis were identified during this process and resolved during team meetings.

Triangulation of quantitative and qualitative data involved summarizing each dataset, identifying convergences and divergences, and integrating results to create a holistic picture of the causes and consequences of Elders not having a healthcare provider. After examining quantitative and qualitative results separately, researchers created a matrix for the side-by-side comparison of each dataset related to access to, and satisfaction with, healthcare (**Table 2**). The matrix detailed areas of (1) *convergence* (i.e., whether results provide the same answer to questions), (2) *expansion* (i.e., whether unanticipated findings of one dataset can be explained by findings in the other), and (3) *complementarity* (i.e., whether qualitative results can contextualize quantitative results) (33). Summary reports of key themes/issues were shared and discussed with the CAB and a group of healthcare professionals who work with Elders that was convened to consult on the dissemination of study findings. Our results were thus generated through an iterative process of drawing out themes and sharing them with community members for guidance. In addition to this member checking process, other measures to ensure credible and trustworthy analysis included the maintenance of detailed notes to document the evolution of study findings and their interpretation across each stage of analysis (34).

## Results

### Quantitative Results

Of the 96 Elders participating in this study, a third (33.3%) indicated that they did not have someone they considered to be a regular or personal healthcare provider. The survey results reflected both similarities and differences between Elders who indicated that they had a personal healthcare provider and those without one. As shown in Table 1, there were no statistically significant differences between groups based on gender, age, or highest level of education. However, there was a greater representation of persons aged 65 and over among the group with a healthcare provider (60.9%) compared to the group without a provider (43.7%). Income levels and internet usage were higher among those reporting having a personal healthcare provider.

**Table 1:** Survey response comparisons between American Indian Elders with and without a personal healthcare provider.

	**No personal healthcare provider** **(*n* = 32)**	**Has a personal healthcare provider** **(*n* = 64)**	**Statistical *p*-value**
**Participant characteristics**			
Female	65.6%	73.4%	0.427
Age 65 or older	43.7%	60.9%	0.110
Associates degree or higher	46.9%	35.9%	0.302
Income of at least $12,000 per year	43.8%	62.7%	0.082^∧^
Uses the Internet	37.5%	57.8%	0.061^∧^
Health self-rated as Fair/Poor	37.5%	29.7%	0.440
**Healthcare access**			
Had health insurance during the previous year	100.0%	100.0%	1.000
Have a usual place to go when sick or needing advice about health	87.5%	93.8%	0.296
Uses the IHS or other tribally operated program for healthcare	87.5%	89.1%	0.821
In the previous 12 months, had trouble finding a doctor or provider who would see them	20.0%	7.0%	0.087^∧^
In the previous 12 months, delayed care due to waiting too long at the healthcare facility	34.4%	15.6%	0.036^*^
In the previous 12 months, did not get a prescription due to cost	18.8%	4.7%	0.026^*^
In the previous 12 months, received an unexpected medical bill after getting care	34.4%	18.8%	0.099^∧^
**Healthcare satisfaction**			
In the previous 12 months, doctors or other health professionals always listened carefully and explained things in a way they could understand	28.6%	71.4%	<0.001^***^
In the previous 12 months, very satisfied with the quality of medical care received	9.4%	60.9%	<0.001^***^
Extremely/very confident they could get care if they needed it	40.6%	77.8%	<0.001^***^

∧ *Statistically significant at p < 0.10*.

* *Statistically significant at p < 0.05*.

*** *Statistically significant at p < 0.001*.

The proportion of Elders reporting that they had “fair” or “poor” health was relatively similar between those without a provider (37.5%) and those with one (29.7%). All Elders had health insurance during the previous year, and almost all reported that they had a usual place to go for healthcare (87.5% among Elders with no personal healthcare provider and 93.8% for Elders with one). Approximately 90% of Elders across both groups received healthcare at the IHS or another tribally operated program.

Differences emerged between the two groups regarding the extent to which they experienced some barriers to accessing healthcare. Persons without a provider were more likely to report difficulty finding doctors willing to see them, delaying care due to long waits at healthcare facilities, forgoing medical prescriptions due to cost, and receiving unexpected medical bills.

Differences between the two groups were also evident in their healthcare-related perceptions and satisfaction. More than two-thirds (71.4%) of Elders with a personal healthcare provider reported that medical professionals always listened to their concerns and explained things in a way that they could understand. In contrast, only 28.6% of Elders without a personal healthcare provider reported this perception. There was an even wider margin between the two groups regarding their level of satisfaction with the quality of medical care they had received during the prior 12 months. Of those with a personal healthcare provider, 60.9% indicated they were very satisfied, compared to only 9.4% of Elders without one. Finally, while 77.8% of Elders with a provider were either extremely or very confident that they could get care if they needed it, fewer than half (40.6%) of Elders without a personal healthcare provider reported such high levels of confidence in their ability to access needed care.

### Qualitative Results

Interviews with Elders who reported that they did not have someone they thought of as their personal healthcare provider revealed the common and often interconnected themes of: (1) good health and self-reliance, (2) persistent bureaucratic and contextual barriers to care, and (3) fear and distrust of doctors and medical care, often stemming from past experiences.

#### Good Health and Self-Reliance

About a third of the Elders who reported not having a personal healthcare provider tied this lack in part to being in generally good health and being able to take care of themselves. A 64-year-old woman commented, “I would make an appointment if I do need it, but if I don't feel like I need an appointment, then I won't.” A 57-year-old man echoed the same sentiment, “I guess when I'm actually real sick and if I can't cure myself from taking over-the-counter, then I guess I will have to go see a real doctor.... But it hasn't [happened], knock on wood.” Several Elders detailed the many ways that they took care of their own health, from drinking lots of water and eating healthy foods, to going for bike rides and chopping wood, to checking their blood pressure and glucose levels regularly. Others described relying on traditional medicine, home remedies, over-the-counter medicines, or alternative approaches (e.g., energy healing) to maintain their health. Many of these individuals explained that they trusted themselves to take care of their health. For example, when asked how likely he is to follow instructions from a doctor about his health, an 83-year-old man asserted, “I don't strictly go by what the doctor tells me. He tells me to swallow a frog or whatever, I don't do that. I just take it [medicine] the way I feel like.”

In addition, of the Elders who did not have a personal healthcare provider, nearly two-thirds reported that they took care of themselves if they were sick, went to the doctor by themselves, and/or did not talk to anyone else about their healthcare decisions. For some, this was because they did not have anyone else to care for them. For example, when asked if there was anyone who cared for her at home when she was sick or needed help, one 60-year-old woman replied, “Never! I was there by myself when I came home from my surgery.... No, my kids don't care.” Another man stated, “I don't have anybody.” Others simply expressed a preference for taking care of themselves, such as a woman in her sixties who explained that she did not talk about her health with anyone because, “I know me better than anybody else.”

#### Barriers to Care

Although some Elders lacked a personal healthcare provider because they did not feel that they needed one, the most common reasons that Elders gave for lacking a provider were related to obstacles within the healthcare system that made it difficult for them to find and establish a relationship with a provider. Several Elders described being deterred from getting care by long wait times. For example, one 59-year-old woman commented, “It takes too long to see a doctor, and my thing is that I just don't like to wait that long. So maybe that's one of the reasons why I don't really like to go, because it's just a waste of time.” Others recalled times when they had been forced to wait, like one 60-year-old woman who explained, “It's hard up there [at the IHS hospital] because then you're sitting in the waiting room, waiting, waiting, waiting, and then you get called into the back and then you sit there. I sat in the back for 5 h one time.” Elders also described struggles making and keeping appointments with a doctor, such as trouble getting through on the phone. A man in his late fifties complained that at his local IHS facility,

They don't tell you when they're going to give you this appointment. All of a sudden, it arrives in the mail. Something comes up and then when it does you're going to have to cancel the visit and they're pretty swamped up at the facility because we don't have enough doctors over there.... Then maybe you're pushed back another month. You're back down to the bottom and then try to climb your way back up to the top.

Other Elders were deterred from seeking care because they didn't know how to find a doctor they liked or were afraid of what care might cost. For example, a 65-year-old man suggested that he would like to choose a new doctor outside of his IHS service area, but could not afford to do so. He explained, “I think a lot of us, we can go over there [to the local IHS clinic] because we can't afford to go to any other facility. If you go to another facility, you have to pay right then and there. Over here, it's provided.” For rural-dwelling Elders, geographic distance aggravated these barriers. A 69-year-old woman noted that her choice of healthcare providers was limited by geography, commenting, “That's one thing about [the IHS hospital]: it's close, it's just a few miles away compared to [the city].... And I hate driving in [the city].”

One obstacle to having a regular healthcare provider that came up repeatedly during our interviews with Elders was the problem of provider turnover. Echoing others, Elders explained that, because of turnover, “I haven't had a regular doctor at all” and “There's too many doctors coming and going, so you can't really have a certain doctor.” In multiple instances, interview participants and the Elder consultants who assisted with the interviews ended up commiserating about the recent loss of a favorite doctor. In one interview, for example, the 69-year-old woman quoted above mentioned a doctor she had liked and asked, “Did you know he's leaving?... He's really gotten us back on the good health track and he's going to be leaving.” The Elder consultant replied, “Now we're going to lose him, we're back down to square one again.” “We lose the good ones,” the participant concluded.

For many Elders, these barriers overlapped in such a way that establishing a regular relationship with a healthcare provider was simply too difficult. In one exemplary comment, a man in his sixties ran down a list of these and other challenges associated with getting care:

They'll assign you a doctor, but you don't know who it's going to be. Then it won't be for another 2 more months before you can get in. Then you have to go and they have to do a blood test.... Then the next time, we were going to make an appointment and he's leaving.... Then when you go in there, you wait almost 20 min before you even get screened. Then you see the doctor and you're out in 10 min.... Then they'll do a referral for you.... Then maybe another month later, they'll say, “Okay, it's approved.” All those things come about.

#### Fear and Distrust of Doctors and Medical Care

In addition to experiencing systemic and administrative barriers, Elders who did not have a regular relationship with a healthcare provider often expressed an overall sense of fear and distrust of doctors and the prospect of receiving medical care. For example, when asked why she was unlikely to seek medical care, a 64-year-old woman answered, “Sometimes I'm kind of like afraid to really tell them or I can't really explain how I'm feeling or what I think is wrong with me.” She went on to explain, “They [doctors] give me the answers about what they... feel that could be wrong with me, but sometimes, I wonder if that's what's really going on with me.” A man in his eighties commented, “Most of these doctors, they don't want to see you get well. They want for you to be sick. They want you there in the office every time they turn around.” Another woman in her sixties commented, “I really hesitate to go see them [doctors] because they tell you, ‘You're this, you're that.' Like every time I go to [the IHS hospital], they tell me I have high blood pressure.” This woman then connected her fear of visiting the hospital to painful memories of her own mother's health struggles: “Sorry I have to get emotional, but my mother, she took all these medications and I think that they just probably affected all her whole system and I think that's why I just hate to go see a doctor because they'll probably say, ‘You need this for that.”'

In fact, suspicion of being needlessly or excessively medicated was a common sentiment among Elders without a regular healthcare provider. One woman in her sixties stated, “If you complain about something hurting or something that's not feeling right, they have a tendency to give you pain medicine—drug you out and numb you out—instead of trying to find a test or a source that might lead to what is causing that trouble for you,” while another recalled how she refused to take pain medication that was prescribed to her after a surgery because she feared “the side effects and then getting hooked on it and I hear a lot of these stories and I don't think I want to go through that.” A third Elder tied her suspicion of medications to a memory of her late sister:

When she passed away there was a paper, those big paper sacks, my nephew was filling it up with all her medicine. It was completely full of medication, and he goes, “Auntie, go look at all the medicine Auntie takes,” and I told her that one time. I told her, “They're just using you as a guinea pig.” They didn't know what was wrong with her.

A fourth Elder's fear of medication was rooted in his observation of the health disparities affecting American Indian people in general. He explained, “The doctor wanted me to [take it] but I said no because I know that nationwide, a lot of Natives have become diabetic all at once. I just said, ‘I can't get on insulin.' I don't want to get on insulin.”

As many of these quotations attest, the fear and mistrust that made some Elders avoid seeking healthcare were often connected to past trauma. In one exemplary case, a 62-year-old woman related how her painful ovarian cysts had first gone undiagnosed and then were treated with a hysterectomy for which she had not given her permission, a traumatic experience that she described as “the invasion of my life.” A second woman in her early sixties described a time when she was struggling with severe vertigo. After being sent home with “some pills” by one doctor, a second doctor at a different healthcare facility told her she had had a stroke. She recalled, “Then me and my son, we look at each other and he said, ‘Did you know you had a stroke?' I says, ‘No.”' Other Elders linked their reluctance to seek care to similar stories of being ignored, misdiagnosed, or profoundly mistreated by medical professionals.

### Mixed-Method Results

As depicted in Table 2, both quantitative and qualitative findings demonstrate that American Indian Elders who do not have someone that they think of as their personal healthcare provider experience particular challenges related to both healthcare access and their own healthcare perceptions and behaviors. Access barriers stemming from long waits at healthcare facilities and the potential costs of medical care were reflected in both sets of data, while the qualitative data revealed that difficulties making appointments, geographic distance, and provider turnover posed additional barriers. In terms of healthcare behaviors and perceptions, both datasets underscored the dissatisfaction with care and lack of confidence in the availability of care that Elders without a regular healthcare provider expressed. The qualitative interviews contextualized the quantitative findings with rich descriptions of the fears and traumatic experiences in which they were rooted for many Elders, as well as Elders' self-reliance in taking care of their own health.

**Table 2:** Triangulation of data on healthcare access and perceptions among American Indian Elders without a personal healthcare provider.

**Approach**	**Quantitative**	**Qualitative**	**Convergence, expansion, and/or complementarity**
**Healthcare access**			
	Elders without a personal healthcare provider were more likely to report barriers to accessing care, such as trouble finding a provider who would see them and delaying care due to long waits at healthcare facilities.	Elders without a personal healthcare provider described several administrative and contextual barriers to care, including long waits, difficulty making appointments, and geographic distance. Provider turnover was a common and significant obstacle to care.	Convergence of both datasets demonstrated significant barriers to accessing care for Elders. Qualitative data provided complementarity by describing additional obstacles that deterred Elders from establishing a regular relationship with a healthcare provider.
	Elders without a personal healthcare provider were more likely to report cost-related problems with medical care, including not getting a prescription due to cost and receiving unexpected medical bills.	Some Elders without a personal healthcare provider expressed reluctance to seek care due to fear of incurring costs.	Convergence of both data sets showed that the potential costs of medical care presented a challenge for some Elders. The quantitative data provided expansion regarding the specific cost-related barriers (i.e., prescriptions, unexpected medical bills) with which Elders struggled.
**Healthcare perceptions**			
	Elders without a personal healthcare provider were less likely to report that their doctors listened to them and explained things carefully, that they were satisfied with the medical care they had received, and that they were confident that they could get care if they needed it.	Many Elders without a healthcare provider described fear and mistrust of doctors and medical care, often due to past experiences of misdiagnoses, trauma, and neglect affecting both themselves and their families. They often described taking care of themselves and not relying on anyone else to help them with their health. Several Elders also explained that they did not access care because they did not need it.	Convergence of both datasets demonstrated that Elders without a regular healthcare provider were likely to have been dissatisfied with their doctors and the care they received, and to lack confidence in their ability to get care. The qualitative data provided complementarity by revealing that these perceptions were often rooted in past experiences. The qualitative data also provided expansion by revealing that some Elders lacked a healthcare provider because they did not feel they needed one and/or they took of their own health themselves.

## Discussion

This mixed-method study examined the role of a consistent and trusted healthcare provider in the health and healthcare experiences of American Indian Elders, and explored some of the causes and consequences of not having such a provider. Although prior studies provide insight into the relationships between access to care, self-rated health, and the presence of a healthcare provider, this study's triangulation of qualitative and quantitative data provide a rich description of the interpersonal and system contexts that shape these relationships.

The importance of continuity of care is well-documented. Benefits of continuity of care include lower mortality, lower risk of preventable hospitalizations, decreased costs, and improved healthcare satisfaction (2, 3, 6–8). When healthcare systems are faced with high levels of healthcare provider turnover, the resulting challenges with continuity of care impact patient health. Consequences include longer time to needed care for Elder patients, poor relationships with healthcare providers, loss of important information, lower healthcare provider ratings, and decreased patient trust in their healthcare providers (35, 36). The results from our analysis further confirm these concerns and underscore the influence of interrupted continuity of care among the American Indian Elder population. In this study, the presence of a stable, consistent healthcare provider correlated significantly with self-rated measures of health, confidence in getting needed care, access to overall healthcare, and satisfaction with care. Self-rated health is a well-established indicator of an Elder's overall health status and has been correlated with related health metrics, including overall wellbeing and mortality (37–39). Poor access to needed care, lacking a regular healthcare provider, and increased age have all correlated with poor self-rated health scores in prior research, as is supported by this study (38).

This study is unique in the inclusion of a subgroup of American Indian Elders rarely included in health research—Elders without a regular healthcare provider. Notably, our findings show that these Elders did not lack a provider due to not having health insurance or a usual place to go when they needed care. Instead, some refrained from seeking medical care because they were not sick and did not feel the need for care, which are commonly cited reasons that adults lack a relationship with a healthcare provider (4). However, the majority of Elders who reported that they did not have a provider described persistent barriers to care, as well as personal histories impacted by discrimination, trauma, and neglect from within the healthcare system. In this research, experiences of barriers, fear/distrust, and a high level of self-sufficiency (either voluntary or because they had no one else to help them) were interconnected; at least half of the Elders who did not have a personal healthcare provider described some combination of these factors as influencing their decisions about seeking healthcare. Barriers to care—including difficulty making appointments, lack of transportation, and cost of medical care—are pervasive sources of health inequity in this population; analyses of these barriers and recommendations to ameliorate them are detailed in our previous work (16, 17, 20, 36, 40). In addition to these challenges, this analysis also underscores the role of fear, mistrust, and negative associations related to healthcare. Indeed, many American Indian Elders' decisions about healthcare are influenced by personal or collective memories of discrimination, neglect, and abuse at the hands of the U.S. healthcare system, such as the sterilization of American Indian women (23) and unethical medical testing with American Indian children (41). Indeed, medical mistrust and experiences of discrimination and microaggressions are strongly associated with poor healthcare outcomes among American Indians (25, 42–44).

Addressing the barriers to care that hinder the formation of a reliable relationship with a healthcare provider for some American Indian Elders will require multilevel interventions, as we discuss in other publications. Key strategies include increasing health systems navigation to improve overall access to healthcare and connect reluctant patients with healthcare providers, workforce improvements to address turnover and continuity of care, and changes to health policy and funding at the state, tribal, and federal levels to establish and sustain a full complement of healthcare services that Elders need (17, 36, 40). However, the perspectives of Elders in this study also indicate that increasing access to care is not sufficient in itself to overcome the fear and distrust that deters some Elders from seeking care (16). Healthcare providers and administrators, along with tribal leaders, must prioritize outreach efforts to build trust and confidence among Elders, taking into consideration the personal and cultural histories of Elders, along with their specific information needs. Such efforts might include providing space for bidirectional learning between Elders and healthcare providers to foster shared understanding (45), increasing access to social support for Elders who live alone or have no one to help them, and tapping into Elders' perspectives on healthcare to help evaluate and make improvements to local healthcare systems. Along with system changes to increase continuity of care and provider longevity within health systems, these approaches are needed to encourage Elders to establish regular and reliable relationships with a healthcare provider.

## Limitations

The generalizability of these findings may be limited by the sample size, particularly for the group of Elders without a personal healthcare provider. The sample of Elders in this study was also unique in that the majority of Elders used IHS and tribal health services. The sample also included many reservation-dwelling Elders whose experiences may be different from the majority of American Indians who live in urban areas. However, we obtained input from the CAB and a group of Elder healthcare experts to provide context and ensure the accuracy and relevance of our results, which likely mitigated some issues with underrepresentation. Moreover, although our sample of Elders may not have been representative of all American Indian Elders, the fact that a third of participants reported not having a healthcare provider suggests that this is likely not an uncommon situation among Elders in general.

## Conclusion

There is an urgent public health need to address the severe and persistent inequities in health and healthcare access affecting American Indian Elders. This research highlights how these inequities may manifest in disrupted continuity of care with a reliable healthcare provider due to administrative and contextual barriers, as well as fear and mistrust of the healthcare system rooted in decades of neglect and abuse. Efforts to improve health equity for American Indian Elders must tackle these interconnected challenges to ensure that Elders receive the care they need.

## Data Availability Statement

The datasets presented in this article are not readily available because permission to share them must be given by the Southwest Tribal Institutional Review Board and the tribal nations participating in this research. Requests to access the datasets should be directed to etrott@pire.org.

## Ethics Statement

The studies involving human participants were reviewed and approved by the Southwest Tribal Institutional Review Board. The patients/participants provided their written informed consent to participate in this study.

## Author Contributions

EJ and EH analyzed and interpreted the qualitative data and wrote the manuscript. EJ and CW administered the surveys and qualitative interviews. DS and AB analyzed and interpreted the quantitative data. CW and DS conceptualized the study. All authors contributed to the writing of the manuscript.

## Funding

This study was funded by the National Institute on Minority Health and Health Disparities (R01 MD010292 and K99 MD015765). The funding source had no role in the design of this study, its execution, analyses, interpretation of the data, or decision to submit results.

## Conflict of Interest

The authors declare that the research was conducted in the absence of any commercial or financial relationships that could be construed as a potential conflict of interest.

## Publisher's Note

All claims expressed in this article are solely those of the authors and do not necessarily represent those of their affiliated organizations, or those of the publisher, the editors and the reviewers. Any product that may be evaluated in this article, or claim that may be made by its manufacturer, is not guaranteed or endorsed by the publisher.
